# Neuroprotection by MKK7 inhibition in excitotoxicity

**DOI:** 10.1186/2193-1801-4-S1-P53

**Published:** 2015-06-12

**Authors:** Alessandro Vercelli, Tiziana Borsello, Alessandra Sclip, Silvia Biggi, Ivan Enrico Repetto, Sara Cimini, Fabio Falleroni, Simone Tomasi, Riccardo Monti, Noemi Tonna, Federica Morelli, Valentina Grande, Oriano Marin, Fabio Bianco, Daniele di Marino

**Affiliations:** Neuroscience Institute Cavalieri Ottolenghi, Italy; Istituto Mario Negri, Italy

**Keywords:** stroke, JNK, ischemia

Excitotoxicity following cerebral ischemia elicits a molecular cascade, which leads neurons to death. One key molecule of this pathway is c-Jun-N-terminal kinase (JNK), a MAP kinase, which plays both physiological and pathological roles in neurons. We have previously shown that JNK blockade by specific cell permeable peptide inhibitors significantly reduces infarct size and neuronal death. On the other hand, JNK inhibition may have detrimental side effects due to blockade of its physiological function. Here we have designed a new inhibitor, which blocks MKK7, an upstream activator of JNK, which mediates its pathological activation. This inhibitor was designed taking advantage of the growth arrest and DNA damage inducible 45β (GADD45β) ability to bind MKK7, optimizing the essential domain of GADD45β and linking it with a spacer to TAT peptide sequence to penetrate cells. This inhibitor significantly reduces neuronal death in two in vitro models of excitotoxic cell death, one induced by NMDA exposure and the other by oxygen glucose deprivation. We tested the MKK7 inhibitor in vivo, in two models of permanent ischemia, the one obtained by electrocoagulation, and the other by thromboembolic occlusion of the Middle Cerebral Artery. In both models, it blocked MKK7 activation and provided significant protection, significantly reducing the infarct size when injected 30’ before the lesion. In the electrocoagulation model, we also tested the efficacy of the peptide when injected 6h after lesion, obtaining similar protection. Therefore, we showed that it is possible to prevent JNK activation in excitotoxicity by specific inhibition of MKK7, preserving the physiological role of JNK driven by MKK4. Targeting MKK7 could represent a novel therapeutic strategy for several diseases involving JNK activation.

